# Computational Modeling to Explain Why 5,5-Diarylpentadienamides are TRPV1 Antagonists

**DOI:** 10.3390/molecules26061765

**Published:** 2021-03-21

**Authors:** Julio Caballero

**Affiliations:** Centro de Bioinformática, Departamento de Bioinformática, Simulación y Modelado (CBSM), Facultad de Ingeniería, Universidad de Talca, Talca 3460000, Chile; jcaballero@utalca.cl; Tel.: +56-712-418-850

**Keywords:** TRPV1 antagonists, docking, interaction fingerprints, QSAR, 2D autocorrelation, LigRMSD

## Abstract

Several years ago, the crystallographic structures of the transient receptor potential vanilloid 1 (TRPV1) in the presence of agonists and antagonists were reported, providing structural information about its chemical activation and inactivation. TRPV1’s activation increases the transport of calcium and sodium ions, leading to the excitation of sensory neurons and the perception of pain. On the other hand, its antagonistic inactivation has been explored to design analgesic drugs. The interactions between the antagonists 5,5-diarylpentadienamides (DPDAs) and TRPV1 were studied here to explain why they inactivate TRPV1. The present work identified the structural features of TRPV1–DPDA complexes, starting with a consideration of the orientations of the ligands inside the TRPV1 binding site by using molecular docking. After this, a chemometrics analysis was performed (i) to compare the orientations of the antagonists (by using LigRMSD), (ii) to describe the recurrent interactions between the protein residues and ligand groups in the complexes (by using interaction fingerprints), and (iii) to describe the relationship between topological features of the ligands and their differential antagonistic activities (by using a quantitative structure–activity relationship (QSAR) with 2D autocorrelation descriptors). The interactions between the DPDA groups and the residues Y511, S512, T550, R557, and E570 (with a recognized role in the binding of classic ligands), and the occupancy of isoquinoline or 3-hydroxy-3,4-dihydroquinolin-2(1*H*)-one groups of the DPDAs in the vanilloid pocket of TRPV1 were clearly described. Based on the results, the structural features that explain why DPDAs inactivate TRPV1 were clearly exposed. These features can be considered for the design of novel TRPV1 antagonists.

## 1. Introduction

Transient receptor potential vanilloid 1 (TRPV1) is a member of the TRP (transient receptor potential) superfamily of ion channels that are selectively expressed in sensory neurons, specifically in C and Aδ nerve fibers [1]. Its activation by means of voltage, heat, protons, or chemical substances increases the transport of calcium and sodium ions [2]; this process contributes to pain sensation. The essential role of TRPV1 in pain events during thermal hyperalgesia [3], visceral hypersensitivity [4], irritable bowel syndrome [5], or postoperative pain [6] has been contributed to its identification as a target in the treatment of pain.

The most characteristic chemical compound that activates TRPV1 is capsaicin, the pungent component of chili peppers, where its vanilloid group is essential for establishing interactions with residues in a TRPV1-binding pocket that contains the polar residues Y511, S512, R557, and E570 [7]. A wide number of synthesized TRPV1 ligands has been reported in the last few decades that contain other groups instead of a vanilloid moiety, such as catechol-containing structures [8,9], 2,3-dihydro-1,4-benzodioxine derivatives [10], 3-fluoro-4-(methylsulfonylamino)phenyl-containing structures [11], and chalcones [12]. Not all of them are activators; in fact, TRPV1 ligands can be divided into agonists and antagonists [13]. Agonists have been investigated due to their effects on the desensitization of TRPV1, leading to pain relief [14,15]. On the other hand, a diverse series of antagonists have been identified with promising therapeutical applications due to their potential analgesic and anti-inflammatory actions in neuropathic pain [16,17]. 

Several years ago, Saku et al. reported that 5,5-diarylpentadienamides (DPDAs) act as TRPV1 antagonists [18]. They examined the effect of different aromatic groups at the 5-position of dienamides on their activities and developed two series (A and B in this work) with either isoquinoline (series A) or 3-hydroxy-2-oxo-1,2,3,4-tetrahydro-5-quinolyl (series B) attached to the amide NH of the pentadienamide. Among the designed compounds, Saku et al. found that the R enantiomer of compound **B36b**, one of the most active DPDAs, significantly blocked mechanical allodynia in rats in a dose-dependent manner and reversed thermal hyperalgesia in rats with sciatic nerve injury. When the DPDAs were reported, the three-dimensional (3D) structure of TRPV1 was not available yet; therefore, the atomistic interactions between the DPDAs and TRPV1 were not analyzed and described. The structures of the TRPV1 that form complexes with agonists and antagonists were revealed more recently [7], providing the possibility to investigate the binding modes and chemical interactions of other active ligands.

In this work, atomistic models of TRPV1–DPDA complexes were developed to determine the 3D structures, to compare the orientations of the ligands, and to describe the chemical interactions that influence the complementarity between ligands and the residues at the binding site. First, molecular docking was used to obtain models of the structures of the complexes. Subsequently, these models were analyzed using chemometrics strategies to compare the orientations of the antagonists, to describe the recurrent interactions between the protein residues and ligand groups in the complexes, and to construct a mathematical model that was able to explain the structure–activity relationship of the studied compounds. At the end of this report, theoretical models are provided that can be used as supplementary information for the experimental efforts in the design of the DPDAs, with an added value to researchers interested in the rational development of novel TRPV1 antagonists.

## 2. Results

### 2.1. The Docking Poses

The chemical structures of the 64 studied compounds and their IC_50_ values (against human TRPV1, transformed to log(1/IC_50_)) are depicted in Table 1. This dataset contains 28 compounds from series A (with isoquinoline), 29 compounds from series B (with 3-hydroxy-3,4-dihydroquinolin-2(1*H*)-one), and 7 compounds from series C (with other heterocycles). It is known in the literature that TRPV1 ligands are composed of three fragments: the head (heterocycles in the DPDAs), the neck (which is a linker consisting of the hydrophobic part of the pentadienamide in the DPDAs), and the tail (the substituents at position 5 of the pentadienamide in DPDAs). It is expected that TRPV1 ligands could bind to a pocket formed by transmembrane helices, where they adopt a “tail-up, head-down” configuration and the head should be close to the S4–S5 linker [19].

**Figure 1:**
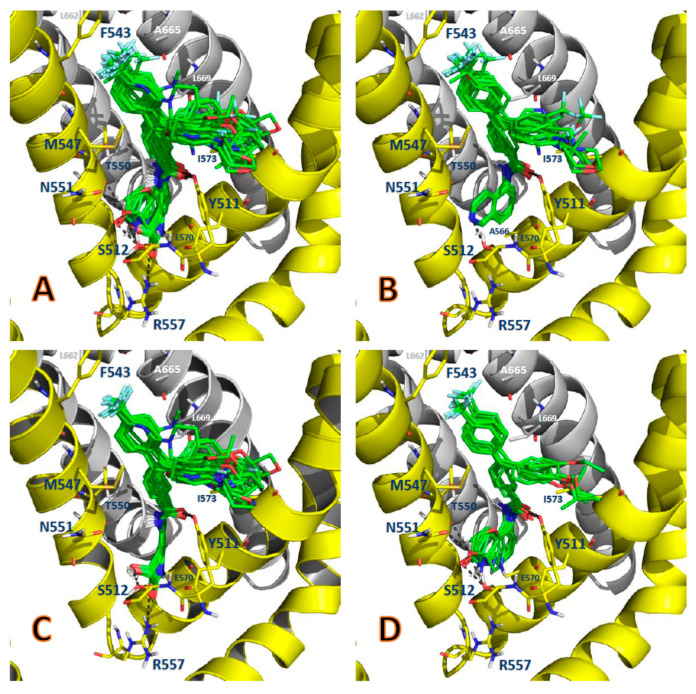
Binding modes of the DPDAs as TRPV1 antagonists. (**A**) Binding modes of the whole dataset. (**B**) Binding modes of the compounds in series A. (**C**) Binding modes of the compounds in series B. (**D**) Binding modes of the compounds in series C. The DPDAs are represented as green sticks, chains A and B of TRPV1 are represented as yellow and gray cartoon representations, respectively. Residues from chains A and B of TRPV1 in the binding site are represented as yellow and gray sticks, respectively.

The docking poses obtained for DPDAs inside the binding pocket of TRPV1 are shown in Figure 1 and are included in the mol2 format as Supplementary Material (SeriesA.zip, SeriesB.zip, and SeriesC.zip files). These results show that orientations were found for all the DPDAs by placing the head, neck, and tail groups at the zones of the TRPV1 binding pocket that typically contain these molecular fragments. Therefore, our docked poses are similar to the pose of capsazepine inside the binding pocket of TRPV1 in the structure with code 5IS0 in the Protein Data Bank (PDB) [7]. However, the orientations of the bicyclic heterocycles (head groups) were not the same for DPDAs from series A and series B, and they also differed from the orientation of the 2,3,4,5-tetrahydro-1*H*-benzo[c]azepine head group in capsazepine. It is also possible to observe that the head and neck groups of DPDAs did not establish the same hydrogen bond (HB) interactions that were observed for capsapezine inside the TRPV1 binding site. A comparison between the poses of DPDAs and capsazepine inside the binding site of TRPV1 is shown in the Supplementary Materials (Figures S1 and S2). The Glide XP (extra precision) scoring energies are reported in Table 1. It is well known in the literature that docking methods are unreliable for calculating binding energies and these values do not correlate well with experimentally determined binding affinities [20]. In this study, a correlation between the Glide XP scoring energies and the experimental log(1/IC_50_) values was found with a low correlation coefficient of *R*^2^ = 0.044.

### 2.2. Comparison between the Poses

To measure the similitude in orientations, the root mean square deviation (RMSD) values for the docked structures were calculated relative to selected references by using LigRMSD [21]. The references were compound **A11b** for series A and compound **B11ac** for series B and C because these compounds contain a 4-(OMe)-phenyl substituent at position cis 5 of the pentadienamide, which is a simple fragment that is present in the topology of the majority of the remaining compounds.

**Figure 2:**
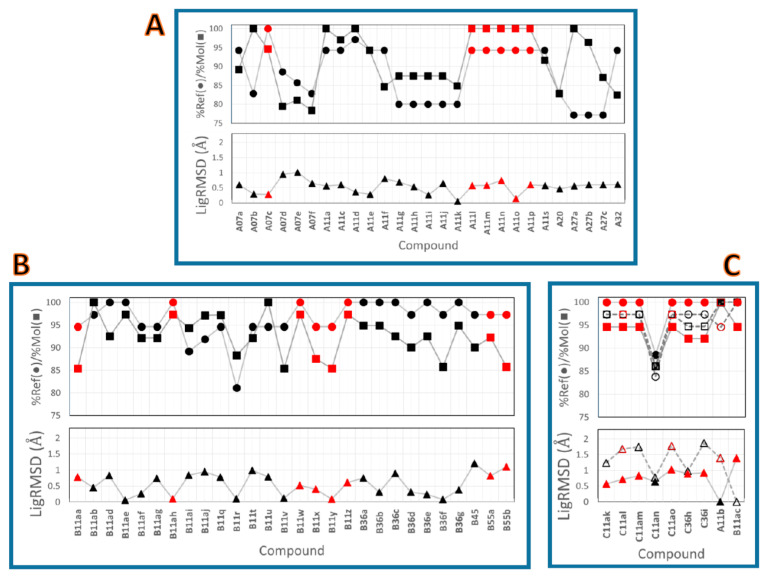
LigRMSD values for compounds from series A (**A**), B (**B**), and C (**C**). The LigRMSD values are represented by triangles, the %Ref match values are represented by circles, and the %Mol match values are represented by squares. Red markers indicate that the flexible mode was used for the LigRMSD calculation. (**C**) also includes LigRMSD, %Ref match, and %Mol match values for **A11b** and **B11ac**. In (**C**), closed triangles, circles, and squares represent LigRMSD, %Ref match, and %Mol match values relative to the reference **A11b**, respectively, and open triangles, circles, and squares represent LigRMSD, %Ref match, and %Mol match values relative to the reference **B11ac**, respectively.

LigRMSD values (relative to the defined references) for the studied compounds are reported in Figure 2. It is noteworthy that LigRMSD < 1.00 Å for compounds from series A (Figure 2A) and LigRMSD < 1.20 Å for compounds from series B (Figure 2B) show that the same binding modes were found. %Ref and %Mol match values >75% indicated that the LigRMSD values were calculated by comparing the coordinates of very similar graphs for both series. Several compounds were compared with the references by considering the flexible mode of LigRMSD. For instance, N atoms at pyridinyl or pyrimidin-5-yl groups for compounds **A07c**, **A11l**, **A11n**, **B11aa**, **B11ah**, **B11w**, etc., were considered identical to C atoms of the phenyl substituent in the reference compounds.

The LigRMSD values for compounds from series C were calculated relative to both references (**A11b** and **B11ac**). These values, reported in Figure 2C, depict that the binding poses of compounds from series C were more similar to the reference **A11b** (lower LigRMSD values ≤ 1 Å). The flexible mode was required for comparing almost all these compounds with **A11b**. Their %Ref and %Mol match values were >92%, with the only exception being compound **C11an** (which was the only compound that contained a 5,6-ring-fused heterocycle instead a 6,6-ring-fused heterocycle as the head). Figure 2C also shows that the LigRMSD value between **A11b** and **B11ac** was 1.4 Å. 

In general, the LigRMSD values were below 2 Å for all the comparisons presented in Figure 2. This point, which is clearly in agreement with the representation of the binding poses in Figure 1B,C, reflects a greater consistency in the docked poses for our studied DPDAs.

**Figure 3:**
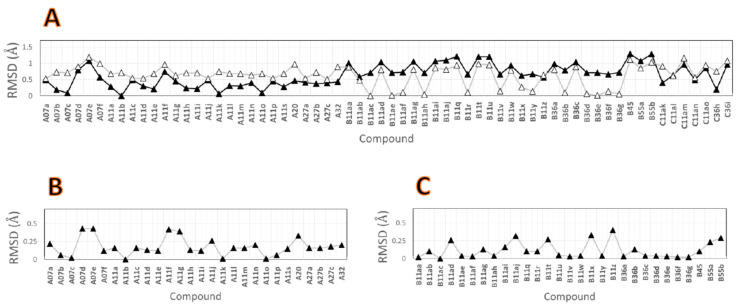
The root mean square deviation (RMSD) values that were used to compare the 3D positions of the neck and head groups of the studied DPDAs. (**A**) RMSD values between the neck groups of compounds relative to the neck groups of **A11b** (closed triangle) and **B11ac** (open triangle). (**B**) RMSD values between the head group of compounds from series A relative to the head group of **A11b**. (**C**) RMSD values between the head group of compounds from series B relative to the head groups of **B11ac**.

An additional analysis was performed to check whether the neck groups of the studied compounds occupied a similar 3D space in the TRPV1 binding site. For this, the coordinates of the neck pentadienamide groups of **A11b** and **B11ac** were selected as references. The RMSD values for the studied compounds relative to these references were calculated and are reported in Figure 3A. It is possible to observe that the majority of the neck groups of the compounds from series A had RMSD values that were below 0.5 Å relative to the neck group of **A11b**, but they had RMSD values between 0.5 and 1.2 Å relative to the neck group of **B11ac**. On the other hand, almost half of the neck groups of the compounds from series B had RMSD values below 0.5 Å relative to the neck group of **B11ac**, but they had RMSD values between 0.5 and 1.3 Å relative to the neck group of **A11b**. Regarding the compounds from series C, it was noted that the coordinates of the neck groups of compounds **C11ak** and **C36h** were closer to the ones for the neck group of **A11b**, and the coordinates of the neck group of the remaining compounds from series C similarly deviated from the coordinates of both references.

In general, despite the differences noted between the binding poses of the compounds from series A and B, the neck pentadienamide group was placed at the same position for the whole set, with RMSD values below 1.3 Å. 

The same analysis was done to verify that the head groups of the studied compounds occupied a similar 3D space at the TRPV1 binding site. For this, the coordinates of the isoquinoline head group of **A11b** and the 3-hydroxy-3,4-dihydroquinolin-2(1*H*)-one head group of **B11ac** were selected as references. The RMSD values for compounds from series A and B relative to these references were calculated and are reported in Figure 3B,C. It is possible to observe that the head groups of compounds from series A had RMSD values below 0.43 Å relative to the head group of **A11b**, and the head groups of compounds from series B had RMSD values below 0.4 Å relative to the head group of **B11ac**. These RMSD values reflect that the head groups had only one orientation for each series A and B. 

### 2.3. Interactions with Residues at the TRPV1 Binding Site

The LigRMSD results found when comparing the positions of the congeneric ligands or their identical or similar fragments show a more detailed analysis that confirmed the similar orientation of the studied compounds obtained via the docking protocol used in this work. Another part of the analysis consisted of the annotation of the recurrent interactions that were observed between the docked ligands and the TRPV1 binding site, which was carried out by computing the interaction fingerprints (IFPs) [22,23].

**Figure 4:**
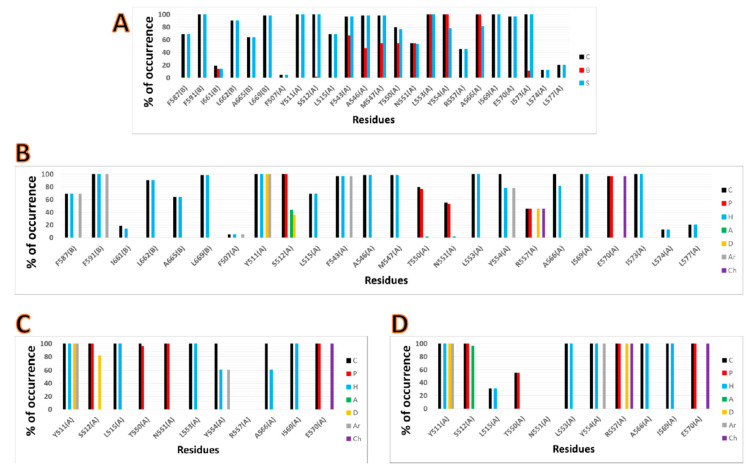
Occurrences of the interaction types at the TRPV1–ligand binding interface for the complexes obtained via docking. The percentages of occurrences of contacts C, interactions with the backbone of the residue B, and interactions with the side chain of the residue S for the 64 complexes (**A**). The percentages of occurrences of chemical interactions: contacts C, polar P, hydrophobic H, HBs where the residue is acceptor A, HBs where the residue is donor D, aromatic Ar, and electrostatic with charged groups Ch for the 64 complexes (**B**), for compounds from series A (**C**), and compounds from series B (**D**).

The IFPs, which were calculated by considering the complexes formed by our 64 docked structures, are reported in Figure 4. Twenty-four TRPV1 residues had contacts with the studied DPDAs, and 20 of them had contacts with more than 40% of the ligands. The TRPV1 channel has four subunits, and the capsaicin binding site is located between two neighboring subunits [7]. The most important residues involved in the interactions with ligands are located at the S2–S3 linker (Y511 and S512), S3 (L515), S4 (F543, A546, M547, T550, N551, L553, Y554, and R557), and the S4–S5 linker (A566, I569, E570, and I573) of one subunit (named A here), and S5 (F587 and F591) and S6 (L662, A665, and L669) of an adjacent subunit (named B here).

The plots of the percentages of occurrences obtained from the IFP calculations in Figure 4A,B show that the residues F591, L662, and A665 in subunit B and the residues F543, A546, and M547 in subunit A had hydrophobic contributions in more than 60% of the docked structures. These residues defined the limits of the hydrophobic pocket that contained the groups at position 5 trans of the DPDAs. It is pertinent to note that M547 was the only residue identified by IFPs that was different between human and rat TRPV1 channels; M547 in rat TRPV1 is replaced by L547 in human TRPV1. Therefore, this residue should be the only one responsible for the different antagonistic activities of DPDAs against rat and human TRPV1s, as reported by Saku et al. [18].

The residue L669 in subunit B and the residues Y511 and I573 in subunit A were identified using IFPs and defined the limits of the hydrophobic pocket that contained the groups at position 5 cis of the DPDAs. The plots of the percentages of occurrences show that these residues had hydrophobic contributions in more than 98% of the docked structures. Interestingly, several of the most potent DPDAs had large groups at position 5 cis of the pentadienamide that established contacts with the backbone of I573. The residue F587 in subunit B was identified using IFPs since it had hydrophobic interactions with the aliphatic hydrocarbon part of the neck pentadienamide group (in 68.8% of the docked complexes), and the residue T550 in subunit A was identified using IFPs since it had polar interactions with the NH of the same group (in 76.5% of the docked complexes). The residue Y511 in subunit A had a special role according to the IFPs: it had the abovementioned role in defining the limits of a hydrophobic pocket and it acted as an HB donor to the CO of the neck pentadienamide group in all the studied DPDAs. 

All the residues that had contacts with the head groups of the DPDAs were in subunit A. The IFP plots of the percentages of occurrences show that the residues L515, L553, Y554, A566, and I569 had hydrophobic contributions in more than 65% of the docked structures, and the residues S512, N551, R557, and E570 had polar contributions in more than 45% of the docked structures. Specifically, S512 and R557 acted as HB donors in 35.9 and 45.3%, respectively, of the docked compounds, and S512 also acted as an HB acceptor in 43.75% of them.

To gain more insight into the specific interactions of the head groups of series A and B, the plots of the percentages of occurrences obtained from the IFP calculations are presented for the residues involved in the interactions with the head groups and amide of the neck group for compounds from series A (Figure 4C) and compounds from series B (Figure 4D). These plots reflect some differences between the polar interactions of the head isoquinoline and 3-hydroxy-3,4-dihydroquinolin-2(1*H*)-one groups of the DPDAs in the vanilloid pocket of TRPV1. More than 95% of the compounds from series A had polar interactions with T550, but only 55.1% of compounds from series B had this interaction. These values reflect that the amide NH groups of the pentadienamide in the large majority of compounds from series A were closer to the polar OH group of T550, but there was a lower proportion of compounds from series B that meet this criterion. Only compounds from series A had polar interactions with the residue N551. At the same time, only compounds from series B had polar interactions with the residue R557, which was hydrogen-bonded to the CO of the 3,4-dihydroquinolin-2(1*H*)-one head groups. Finally, Figure 4C shows that S512 acted as an HB donor in 82.1% of the compounds from series A, forming this interaction with the N of isoquinoline; meanwhile, Figure 4D shows that S512 acted as an HB acceptor in 96.6% of the compounds from series B, forming this interaction with the OH of 3-hydroxy-3,4-dihydroquinolin-2(1*H*)-one. 

A summary of the role of TRPV1 residues in HB interactions with compounds from series A and B, and their role in specific HB interactions with the head groups of compounds from series C are listed in Table 2. According to the IFPs, S512 acted as a donor in HBs with CO groups of 2-oxo-1,2-dihydro-quinoline (**C11al**), 3,4-dihydroquinolin-2(1*H*)-one (**C11am** and **C36i**), indolin-2-one (**C11an**), and 2*H*-benzo[b]-[1,4]oxazin-3(4*H*)-one (**C11ao**). It also acted as an HB donor with the OH group of 1,2,3,4-tetrahydroquinolin-3-ol in **C11ak**, and the same group in this compound formed an additional HB with the side chain CO of N551. The IFPs identified that compound **C36h**, which also contained a 1,2,3,4-tetrahydroquinolin-3-ol group, formed an HB between its OH group and the backbone CO of T550.

### 2.4. Docking Models Explain Why DPDAs are TRPV1 Antagonists

Recently, Gao et al. reported the TRPV1 structures (in lipid nanodiscs) forming complexes with the antagonist capsazepine (PDB code 5IS0) and the agonist resiniferatoxin (PDB code 5IRX) [7]. They found that the TRPV1 vanilloid pocket had conformational changes induced by antagonist and agonist effects. The agonist resiniferatoxin coordinates the formation of a salt bridge between R557 and E570, leading to a movement of the S4–S5 linker that facilitates the opening of the lower gate of TRPV1; meanwhile, capsazepine prevents this salt bridge formation leading to the closed state of TRPV1. 

**Figure 5:**
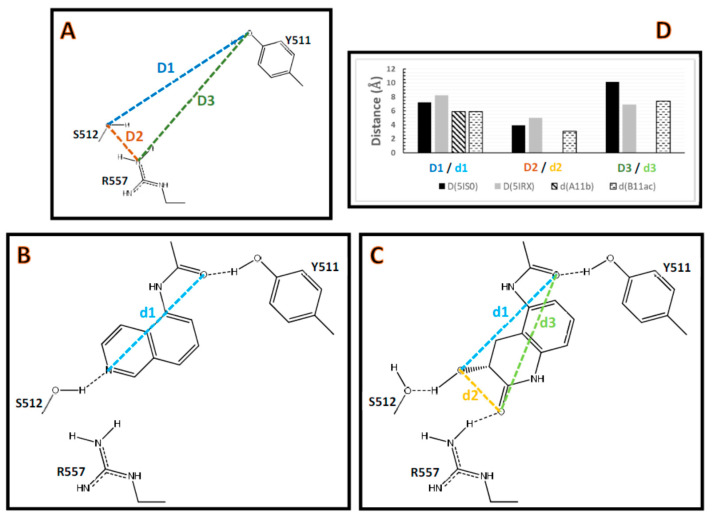
Distances *D* between the residue atoms involved in the HBs and the distances *d* between the ligand atoms involved in the HBs. (**A**) Definitions of the distances *D1*, *D2*, and *D3* in the TRPV1 structure. (**B**) Definition of the distance *d1* in the compounds from series A. (**C**) Definitions of the distances *d1*, *d2*, and *d3* in the compounds from series B. (**D**) Values of distances *D* in the structure prepared from the Protein Data Bank (PDB) with code 5IS0 for docking calculations (the original PDB formed a complex with the antagonist capsazepine) and in the structure from the PDB with code 5IRX (which formed a complex with the agonist resiniferatoxin); values of the distances *d* in the docked poses of compounds **A11b** and **B11ac** representing compounds from series A and B are also included.

To show the differences between TRPV1 conformations when an antagonist or agonist is present, the distances *D* were measured in the vallinoid pocket of the structures with codes 5IS0 and 5IRX (Figure 5). *D1* is the distance between the oxygen from the side chain OH of Y511 and the oxygen from the side chain OH of S512, *D2* is the distance between the oxygen from the side chain OH of S512 and the most exposed Nη of R557, and *D3* is the distance between the oxygen from the side chain OH of Y511 and the most exposed Nη of R557 (Figure 5A). The distances *D1*, *D2*, and *D3* in the PDB with code 5IS0 are significantly different when compared to those distances in the PDB with code 5IRX (Figure 5D).

Since DPDAs are antagonists, docking calculations were performed inside the TRPV1 structure that contained the antagonist capsazepine (PDB code 5IS0). The results showed only one orientation for the head and the pentadienamide amide groups for compounds from series A forming HBs with Y511 and S512, and only one orientation for the head and the pentadienamide amide groups for compounds from series B forming HBs with Y511, S512, and R557. The distances *d* were measured in the reference compounds **A11b** and **B11ac**, representing compounds from series A and B, respectively (Figure 5B,C), where *d1* is defined as the distance between the CO oxygen of the amide and the N of the isoquinoline in compound **A11b** and the distance between the CO oxygen of the amide and the OH oxygen of 3-hydroxy-3,4-dihydroquinolin-2(1*H*)-one in compound **B11ac**, *d2* is the distance between OH and CO oxygen atoms in 3-hydroxy-3,4-dihydroquinolin-2(1*H*)-one, and d3 is the distance between the CO oxygen atom in 3-hydroxy-3,4-dihydroquinolin-2(1*H*)-one and the amide CO oxygen atom (*d2* and *d3* were only defined for compound **B11ac**).

The docking results suggest that the *D* and *d* distances were optimal for the binding of the DPDAs in the TRPV1 structure with code 5IS0, but they are not optimal in the structure with code 5IRX. It is worth noting that the *d1* distances had the same value of 5.9 Å for both reference compounds from series A and B. For the TRPV1 structure with code 5IS0, a comparison between the *D* and *d* distances showed that *D1* > *d1*, *D2* > *d2*, and *D3* > *d3*. However, *D3* < *d3* for the TRPV1 structure with code 5IRX. Empirically, it could be reasonable to suppose that the *d* distances (defined with atoms of the ligand that formed the HBs) should be less than the *D* distances (defined with atoms of the protein that formed the HBs). This empirical rule was only fulfilled for the structure with code 5IS0 (which contained the antagonist capsazepine). 

To verify that the TRPV1 structure with code 5IRX (the conductive form of TRPV1) was not optimal for binding DPDAs, docking calculations were performed by using this structure as a receptor. These poses and the comparison between them using LigRMSD are presented in the Supplementary Materials (Figures S3 and S4). The resulting poses for the majority of compounds had HBs with residues in the vanilloid pocket, but the formation of several HBs were not satisfied at the same time. It was also possible to observe higher RMSD values between the neck and head groups in these poses, which reflected a lower homogeneity in the obtained conformations and different HB interaction patterns. 

Therefore, the docking results shown here represent a model of the mechanism of action of DPDAs as TRPV1 antagonists: they prevented the formation of a salt bridge between R557 and E570 when binding to the vanilloid pocket, just as capsazepine does [7], not allowing the opening of the TRPV1 channel.

### 2.5. 2D Autocorrelation Models for Describing Differential Activities

The different potencies of DPDAs as TRPV1 antagonists were found using a quantitative structure–activity relationship (QSAR) model that contained only topological information. After calculating the 2D autocorrelation descriptors and applying a variable selection protocol, the best model describing the linear relationship between log(1/IC_50_) included six descriptors, as shown in Equation (1):

(1) $$\begin{array}{l} \log\left(1/{\mathrm{IC}}_{50}\right)=4.76\times \mathrm{ATS}1m+32.3\times \mathrm{MATS}1e+6.42\times \mathrm{MATS}7e\\ \;+3.07\times \mathrm{MATS}8e+14.9\times \mathrm{MATS}1p-5.62\times \mathrm{GATS}5e-12.7, \end{array}$$

*N* = 52, *R*^2^ = 0.674, *s* = 0.586, *p* < 10^−5^, *F* = 15.5, *Q*^2^ = 0.549, *s*_CV_ = 0.689.

In Equation (1), *N* is the number of compounds in the training set, *R^2^* is the square of the correlation coefficient, *s* is the standard deviation of the regression, *p* is the significance of the regression model, and *Q*^2^ and *s*_CV_ are the correlation coefficient and standard deviation of the leave-one-out cross-validation (LOO-CV), respectively. 

The best model explained 67.4% of the TRPV1 antagonistic activity variance. A value of *Q*^2^ > 0.5 reflects the importance of each member of the training set to a complex relationship [24]. The model included one Broto–Moreau’s coefficient (ATS1m), four Moran’s indices (MATS1e, MATS7e, MATS8e, and MATS1p), and one Geary’s coefficient (GATS5e); no significant intercorrelation between these descriptors was found. The model included three weighted terms with an influence on the potency of DPDAs as TRPV1 antagonists. It showed a positive effect of an atomic-mass-weighted term due to only one descriptor, a positive effect of an atomic polarizability weighted term due to only one descriptor, and a complex effect of atomic Sanderson electronegativity weighted terms due to four descriptors; the van der Waals volume-weighted terms had no effect. The predictions of the training set compounds are found in Table 1. The analysis of the residuals shows that nearly two thirds of the compounds had residuals below 0.5, eight compounds had residuals between 0.5 and 0.75, eight compounds had residuals between 0.75 and 1.0, and the compound **B11ab** had a residual of 1.5. The outlier behavior of **B11ab** can be explained by considering the structural similarity of compounds **B11ab**, **B11ac**, and **B11ad** and their different log(1/IC_50_) values (log(1/IC_50_) = −1.519 for **B11ab** and log(1/IC_50_) > 0.5 for **B11ac** and **B11ad**).

**Figure 6:**
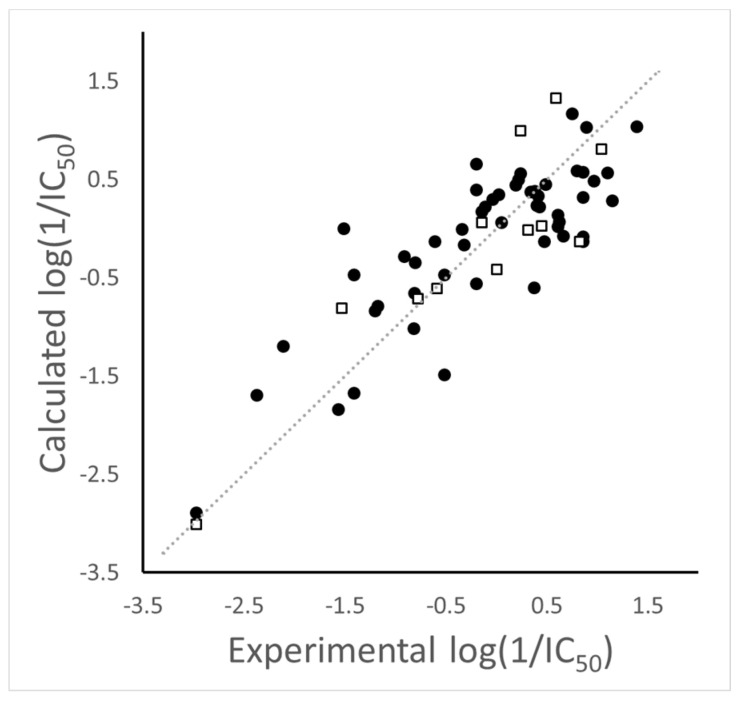
Scatter plot of the predicted versus experimental log(1/IC_50_) values for the training set (●) and the test set (□) using the 2D autocorrelation model.

The model adequately predicted the activities of 12 compounds in a test set; the correlation of this prediction test was *R*^2^_test_ = 0.788 (compounds included in the test set and their predictions are also in Table 1). Plots of the training and test set predictions versus the experimental log(1/IC_50_) values are shown in Figure 6. Additional tests were used to evaluate the quality of the model. First, six replicas of the model with different training–test splits were done and averaged values of *R*^2^ = 0.694, *Q*^2^ = 0.578, and *R*^2^_test_ = 0.706 were obtained (Table S1 in the Supplementary Material). Then, six random reorganization tests were performed and a clear deterioration of the *R*^2^, *Q*^2^, and *R*^2^_test_ values was obtained (Table S2 in the Supplementary Materials).

Since the 2D autocorrelation descriptors have no direct interpretation, the derived model should be considered as a mathematical abstract relationship with no mechanistic interpretation. However, the weighted terms represent the chemical properties that are related to the difference in the potency of compounds and the definition of lags leads to different mathematical schemes that represent different topological interpretations of the molecules under study. 

## 3. Materials and Methods 

### 3.1. Dataset 

Table 1 contains the structural representations of the studied DPDAs. In this report, compounds that contained isoquinoline were from series A and compounds that contained 3-hydroxy-3,4-dihydroquinolin-2(1*H*)-one were from series B. Each compound was named using the defined series followed by the identification given for the compound in the paper of Saku et al. [18]. The few compounds that contained other heterocycles were grouped into series C: compounds that contained 1,2,3,4-tetrahydroquinolin-3-ol were named **C11ak** and **C36h**, the compound that contained 2-oxo-1,2-dihydro-quinoline was named **C11al**, compounds that contained 3,4-dihydroquinolin-2(1*H*)-one were named **C11am** and **C36i**, the compound that contained indolin-2-one was named **C11an**, and the compound that contained 2*H*-benzo[b]-[1,4]oxazin-3(4*H*)-one was named **C11ao**.

Only R enantiomers were considered for compounds forming racemic mixtures (compounds from series B, **C11ak**, and **C36h**). This assumption was plausible after taking into account the fact that Saku et al. observed not too different IC_50_ values when R and S enantiomers of compounds **B11ae** and **B36b** were evaluated [18]. Therefore, they suggested that the TRPV1 antagonistic activities of DPDAs are only slightly influenced by this effect.

The experimental IC_50_ values were taken from the paper of Saku et al. [18], where they were based on the inhibition of the capsaicin-induced influx (100 nM) of Ca^2+^ into human TRPV1-expressing 293 Epstein−Barr virus nuclear antigen cells. The structures were sketched in Maestro’s molecular editor (Maestro 10.2.011, Schrödinger LLC, New York, NY, USA, 2015). The resultant dataset of 64 compounds was then processed using Maestro’s module LigPrep (the protonation states of the ionizable groups were calculated and defined at physiological pH).

### 3.2. Molecular Docking Calculations

The Glide method from Schrödinger suite was used for performing the docking calculations [25]. The coordinates of TRPV1 in the PDB structure with ID 5IS0 (the complex of TRPV1 from *Rattus norvegicus* with the antagonist capsazepine, solved at a 3.43 Å resolution) were used for constructing the receptor model. The protein structure was prepared by using the Protein Preparation Wizard tool from Maestro (Protein Preparation Wizard, Schrödinger LLC, New York, NY, USA, 2015), including the bond order assignments, additions of hydrogen atoms, and predictions of protonation states of the charged residues. Molecular minimization of the protein system was performed by using the Impact refinement module [26] and the OPLS3 force field [27] with heavy atoms restrained with a harmonic potential of 25 kcal mol^–1^ Å^–2^ and unrestrained hydrogens (convergence was reached when the RMSD was below 0.30 Å).

A grid box of 30 × 30 × 30 Å^3^ was centered on the center of mass of capsazepine to cover the whole binding site. The Glide standard (SP) and extra (XP) precision modes were used in the docking calculations, where the parameters were set as described in previous applications [28,29,30]. The poses were selected based on the lower Glide scoring energy (by considering the top five scoring positions), the requirement of the similarity of the orientations (poses that had the head outside the vanilloid pocket were discarded) [31,32], and the reasoning that analog ligands should have similar chemical interactions when groups are conserved [33,34]. 

### 3.3. Comparison of the Binding Poses

It is expected that the binding mode of congeneric compounds should be conserved. Therefore, the binding poses obtained by docking calculations were compared to check this assertion. LigRMSD [21], which is a web server for the automatic matching and RMSD calculations between identical or similar chemical compounds, was used to provide such a comparison. 

LigRMSD calculates RMSD values by considering only the common graphs between molecules (the maximum common substructure). The matching is defined using the %Ref and %Mol match values. %Ref match is the percent of common graphs between the docked compound and a selected reference relative to the total number of atoms of the selected reference. %Mol match is the percent of common graphs between the docked compound and the selected reference relative to the total number of atoms of the docked compound. These values represent the maximal similitude between the compared compounds; therefore, an RMSD value with high %Ref match and %Mol match values is associated with a major resemblance between the compared compounds.

First, the docked poses obtained for the compounds from series A and B were compared with the docked poses obtained for the references **A11a** and **B11u**, respectively. Then, comparisons between molecular fragments were established by using the same compounds as references. LigRMSD strict mode was used by default, while the flexible mode was used when the matching of a pair of different atom types contributed to the comparison. Only heavy atoms were considered in the RMSD calculations.

### 3.4. IFP Calculations

Chemical interactions with a high frequency between the docked poses of ligands and the residues in the TRPV1 binding site were captured using IFPs [35]. IFPs from Maestro (Maestro, Schrödinger LLC, New York, NY, 2015) were used, accounting for polar (P), hydrophobic (H), and aromatic (Ar) interactions. They also detect HBs with an acceptor group (A), HBs with a donor group (D), and electrostatic interactions with charged groups (Ch). An interaction was identified when heavy atoms from a residue and the ligand were within a cut-off distance of 4.0 Å. HBs were defined with a maximum distance between the H and the acceptor (A) heavy atom of 2.5 Å, a minimum donor (D) angle (D–H…A) of 120.0°, and a minimum acceptor angle of (A…H–D) of 90° (default parameters). The interactions were also separated into those with backbone and side-chain functional groups.

### 3.5. 2D Autocorrelation QSAR Modeling

The 2D autocorrelation descriptors defined by Broto–Moreau (ATS, Equation (2)) [36], Moran (MATS, Equation (3)) [37], and Geary (GATS, Equation (4)) [38] were employed for creating a QSAR correlation model that explained the differential activities of the studied DPDAs as TRPV1 antagonists:

(2) $$\mathrm{ATS}\left(p_{k},l\right)=\sum_{i}\delta_{ij}p_{ki}p_{kj},$$

(3) $$\mathrm{MATS}\left(p_{k},l\right)=\frac{N}{2L}\frac{\sum_{ij}\delta_{ij}\left(p_{ki}-{\bar{p}}_{k}\right)\left(p_{kj}-{\bar{p}}_{k}\right)}{\sum_{i}\left(p_{ki}-{\bar{p}}_{k}\right)},$$

(4) $$\mathrm{GATS}\left(p_{k},l\right)=\frac{\left(N-1\right)}{4L}\frac{\sum_{ij}\delta_{ij}\left(p_{ki}-{\bar{p}}_{k}\right)\left(p_{kj}-{\bar{p}}_{k}\right)}{\sum_{i}\left(p_{ki}-{\bar{p}}_{k}\right)}.$$

In Equations (1)–(3), ATS(*p_k_*,*l*), MATS*(p_k_*,*l*) and GATS(*p_k_*,*l*) are the Broto–Moreau’s autocorrelation coefficient, Moran’s index, and Geary’s coefficient, respectively. These descriptors are defined at spatial lag *l* and properties *p_k_*, *p_ki_* and *p_kj_* are the values of the property *k* of atom *i* and *j* respectively, ${\bar{p}}_{k}$ is the average value of the property *k*, *L* is the number of nonzero values in the sum, *N* is the number of atoms in the molecule, and *δ*(*l*,*d_ij_*) is the Dirac delta function defined in Equation (5):

(5) $$\delta \left(l,d_{ij}\right)=\left\{\begin{array}{c} 1\;if\;d_{ij}=l\\ 0\;if\;d_{ij}\neq l \end{array}\right\}.$$

In Equation (5), *d_ij_* is the topological distance (spatial lag) between atoms *i* and *j*.

The abovementioned 2D autocorrelation descriptors were calculated by using DRAGON version 3.0 software (Milano Chemometrics, Milano, Italy, 2003) with information of the interdependence between the atomic *p_k_* properties: atomic masses (*m*), atomic van der Waals volumes (*v*), atomic Sanderson electronegativities (*e*), and atomic polarizabilities (*p*). These properties were connected by lags *l* defined from 1 up to 8 in molecular graphs. 2D autocorrelation descriptors have been extensively applied to create QSAR models with success [39,40,41,42,43,44]. Ninety-six descriptors were computed and those with constant values were discarded. Then, collinearity was checked and the descriptor with the lower variance from each pair of collinear descriptors was eliminated (descriptors with *R*^2^ > 0.90 were considered as collinear). 

The dataset was randomly split into a training set (52 compounds) and a test set (12 compounds). The IC_50_ values against human TRPV1 (in nM) were converted into logarithmic values log(1/IC_50_). A linear genetic algorithm (GA) search was carried out by exploring multiple regression models of the training set in the program BuildQSAR [45]. The initial population for GA included 100 individuals; novel generations were constructed using crossover, single-point mutations, and tournament selection. The GA fitness function was the mean square error of the data fitting and the end of the search was found when 90% of the generations reached the same target fitness score. The best model was selected by considering the *R*^2^ of the fitting (*R*^2^ > 0.8) and the LOO cross-validation (higher *Q*^2^). The final validation of the selected model was done by evaluating the predictive capacities in the test set.

## 4. Conclusions

The orientations and chemical interactions of the DPDAs at the binding site of TRPV1 were studied by using a docking protocol and chemometric strategies to produce an exhaustive description of the difference between poses and the resultant chemical interactions with residues at the binding site. This endeavor increased the confidence in the reported docked poses since this analysis provided specific measures that allowed for comparing the proposed poses of DPDAs with the poses of classic ligands from previous structural information about TRPV1 antagonists.

The role of the residues Y511, S512, T550, R557, and E570 in the binding of TRPV1 agonists and antagonists was discussed in the report of Gao et al. in 2016 [7]. The present report comments on the role of these residues in the binding of isoquinoline and 3-hydroxy-3,4-dihydroquinolin-2(1*H*)-one groups of the DPDAs inside the vanilloid pocket of TRPV1. The homogeneity of the orientations for a great number of compounds and the perfect geometry of the head groups for establishing chemical interactions in the vanilloid pocket (specifically the HBs with some of the abovementioned residues) are aspects that contributed to the confidence in the results presented here. The docking results show that the isoquinoline and 3-hydroxy-3,4-dihydroquinolin-2(1*H*)-one groups of the DPDAs had the exact geometry to establish HBs with the residues Y511, S512, and R557 in the conformation of TRPV1 that contained the antagonist capsazepine (structure with the PDB code 5IS0), and the geometry was not optimal to establish these interactions with these residues in the TRPV1 conformation that contained the agonist resiniferatoxin (structure with the PDB code 5IRX). Therefore, the antagonistic role of the DPDAs was explained by atomistic models reported here. Similar to capsazepine, the DPDAs did not facilitate the formation of a salt bridge between R557 and E570, which is essential for TRPV1 activation [7].

The scoring energies of the docking models did not correlate with the experimental activities; instead, by using 2D autocorrelation descriptors, the QSAR models indicated that a complex relationship contained in the topological information of structures could be used to interpret the different potencies of compounds. Such relationships were presented and validated using standard QSAR internal and external validation strategies.

In general, this work explained why the DPDAs were TRPV1 antagonists (and not agonists). The results could be useful for researchers that want to know more about TRPV1 antagonistic inactivation. Considering that the PDB structure of TRPV1 in the presence of capsazepine (5IS0) is the only one at this moment with a good resolution, the results reported here indicate that this inactive/nonconductive TRPV1 conformation is useful for explaining why other TRPV1 modulators (such as DPDAs) are also antagonists.

## Figures and Tables

**Table 1:** Structures of 5,5-diarylpentadienamides (DPDAs) as transient receptor potential vanilloid 1 (TRPV1) antagonists. Experimental and predicted log(1/IC_50_) values (in nM) using a 2D autocorrelation quantitative structure–activity relationship (QSAR) model and Glide XP (extra precision) scoring energy values.

**Series A**	** 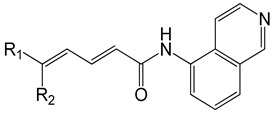 **				
**Compound**	**R_1_**	**R_2_**	**Experimental log(1/IC_50_)**	**Predicted log(1/IC_50_)**	**Glide XP Score (kcal/mol)**
**A07a**	4-(CF_3_)-phenyl	4-(CF_3_)-phenyl	0.377	0.382	−7.75
**A07b**	Phenyl	Phenyl	−1.568	−1.837	−9.10
**A07c**	6-(CF_3_)-pyridin-3-yl	6-(CF_3_)-pyridin-3-yl	−2.114	−1.192	−10.84
**A07d**	4-(OCF_3_)-phenyl)	4-(OCF_3_)-phenyl	−0.820	−1.015	−6.83
**A07e**	4-(tBu)-phenyl	4-(tBu)-phenyl	0.745	1.169	−6.83
**A07f**	3-(CF_3_)-phenyl	3-(CF_3_)-phenyl	−0.204	−0.557	−7.71
**A11a**	4-(CF_3_)-phenyl	Phenyl	0.854	−0.127	−10.85
**A11b ^1^**	4-(CF_3_)-phenyl	4-(OMe)-phenyl	0.824	−0.132	−10.79
**A11c**	4-(CF_3_)-phenyl	4-(F)-phenyl	1.143	0.285	−10.68
**A11d**	4-(CF_3_)-phenyl	4-(OH)-phenyl	−0.914	−0.279	−9.33
**A11e**	4-(CF_3_)-phenyl	3-(CN)-phenyl	−0.322	−0.164	−7.88
**A11f**	4-(CF_3_)-phenyl	4-Morpholinophenyl	0.237	0.560	−7.97
**A11g**	4-(CF_3_)-phenyl	Thiophen-2-yl	0.018	0.350	−10.28
**A11h ^1^**	4-(CF_3_)-phenyl	Thiophen-3-yl	0.310	−0.012	−7.74
**A11i**	4-(CF_3_)-phenyl	Furan-2-yl	−0.519	−0.470	−7.36
**A11j**	4-(CF_3_)-phenyl	Furan-3-yl	−0.519	−1.487	−10.04
**A11k**	4-(CF_3_)-phenyl	5-(Me)-furan-2-yl	0.469	−0.128	−10.59
**A11l ^1^**	4-(CF_3_)-phenyl	Pyridin-3-yl	−1.531	−0.810	−10.70
**A11m**	4-(CF_3_)-phenyl	Pyridin-4-yl	−1.204	−0.837	−10.81
**A11n**	4-(CF_3_)-phenyl	Pyrimidin-5-yl	−2.380	−1.695	−10.48
**A11o**	4-(CF_3_)-phenyl	Cyclohex-1-en-1-yl	0.481	0.452	−10.55
**A11p**	4-(CF_3_)-phenyl	3,6-Dihydro-2*H*-pyran-4-yl	−1.415	−0.467	−9.54
**A11s ^1^**	4-(CF_3_)-phenyl	4-(NMe_2_)-phenyl	0.444	0.027	−11.05
**A20**	4-(CF_3_)-phenyl	4-(CF_3_)-phenyl	−0.613	−0.127	−9.04
**A27a ^1^**	4-(CF_3_)-phenyl	H	−2.973	−3.007	−8.52
**A27b**	4-(CF_3_)-phenyl	Me	−2.978	−2.888	−10.09
**A27c ^1^**	4-(CF_3_)-phenyl	nBu	−0.778	−0.711	−10.88
**A32**	4-(CF_3_)-phenyl	4-(Morpholinomethyl)-phenyl	−0.204	0.663	−4.83
**Series B**	** 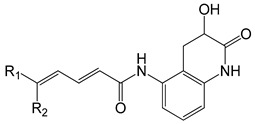 **				
**Compound**	**R_1_**	**R_2_**	**Experimental log(1/IC_50_)**	**Predicted log(1/IC_50_)**	**Glide XP Score (kcal/mol)**
**B11aa**	4-(CF_3_)-phenyl	2-(Piperidin-1-yl)-pyrimidin-5-yl)	0.367	−0.600	−10.41
**B11ab**	4-(CF_3_)-phenyl	4-(OH)-phenyl	−1.519	0.006	−11.32
**B11ac**	4-(CF_3_)-phenyl	4-(OMe)-phenyl	0.602	0.024	−11.85
**B11ad**	4-(CF_3_)-phenyl	4-(OCF_3_)-phenyl	0.854	−0.081	−10.94
**B11ae**	4-(CF_3_)-phenyl	4-(OEt)-phenyl	0.409	0.335	−12.02
**B11af**	4-(CF_3_)-phenyl	2-(OEt)-phenyl	−0.204	0.399	−11.85
**B11ag**	4-(CF_3_)-phenyl	3-(OEt)-phenyl	0.046	0.062	−11.26
**B11ah ^1^**	4-(CF_3_)-phenyl	6-(OEt)-pyridin-3-yl	−0.591	−0.607	−11.98
**B11ai**	4-(Cl)-phenyl	4-(OEt)-phenyl	0.620	0.071	−9.34
**B11aj**	4-(Me)-phenyl	4-(OEt)-phenyl	0.398	0.237	−8.96
**B11q**	4-(CF_3_)-phenyl	4-(F)-phenyl	0.959	0.488	−10.90
**B11r**	4-(CF_3_)-phenyl	Furan-2-yl	−0.806	−0.342	−11.42
**B11t**	4-(CF_3_)-phenyl	4-(NMe_2_)-phenyl	−0.146	0.174	−10.17
**B11u**	4-(CF_3_)-phenyl	Phenyl	0.420	0.222	−10.61
**B11v ^1^**	4-(CF_3_)-phenyl	4-(Piperidin-1-yl)-phenyl	0.585	1.327	−12.59
**B11w**	4-(CF_3_)-phenyl	6-(NMe_2_)-pyridin-3-yl	−0.813	−0.656	−10.22
**B11x**	4-(CF_3_)-phenyl	6-(pyrrolidin-1-yl)-pyridin-3-yl	−0.342	−0.006	−11.63
**B11y**	4-(CF_3_)-phenyl	6-(piperidin-1-yl)-pyridin-3-yl)	0.187	0.447	−12.38
**B11z**	4-(CF_3_)-phenyl	2-(NMe_2_)-pyrimidin-5-yl	−1.415	−1.671	−11.18
**B36a**	4-(CF_3_)-phenyl	4-(O-*n*-Pr)-phenyl	1.097	0.573	−11.86
**B36b**	4-(CF_3_)-phenyl	4-(O-*i*-Pr)-phenyl	0.854	0.581	−11.62
**B36c ^1^**	4-(CF_3_)-phenyl	4-(O-*t*-Bu)-phenyl	1.041	0.806	−10.05
**B36d**	4-(CF_3_)-phenyl	4-Cyclobutoxyphenyl	1.387	1.041	−12.30
**B36e**	4-(CF_3_)-phenyl	4-(Cyclopropylmethoxy)phenyl	0.886	1.034	−12.12
**B36f ^1^**	4-(CF_3_)-phenyl	4-((Tetrahydro-2H-pyran-4-yl)oxy)-phenyl	0.237	0.998	−12.36
**B36g ^1^**	4-(CF_3_)-phenyl	4-(Cyanomethoxy)-phenyl	−0.146	0.060	−12.02
**B45**	4-(CF_3_)-phenyl	4-(Oxetan-3-yloxy)-phenyl	−0.041	0.300	−9.27
**B55a ^1^**	2-(NMe_2_)-6-(CF_3_)-pyridin-3-yl	4-(F)-phenyl	0.000	−0.416	−10.65
**B55b**	2-(Piperidin-1-yl)-6-(CF_3_)-pyridin-3-yl	4-(F)-phenyl	0.215	0.498	−9.96
**Series C**	** 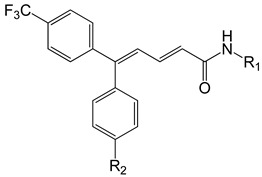 **				
**Compound**	**R_1_**	**R_2_**	**Experimental log(1/IC_50_)**	**Predicted log(1/IC_50_)**	**Glide XP Score (kcal/mol)**
**C11ak**	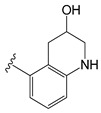	OEt	0.854	0.321	−11.18
**C11al**	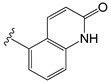	OEt	0.658	−0.076	−10.76
**C11am**	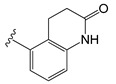	OEt	0.602	0.139	−10.66
**C11an**	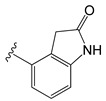	OEt	−0.114	0.224	−11.56
**C11ao**	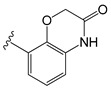	OEt	−1.176	−0.786	−8.27
**C36h**	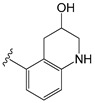	O−*i*−Pr	0.796	0.593	−11.74
**C36i**	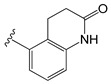	O−*i*−Pr	0.337	0.380	−10.38

^1^ Test set compounds.

**Table 2:** Role of the TRPV1 residues in the HB interactions with head groups and an amide of the neck groups of the DPDAs.

Compound	Head Group	Residues and Their Role in an HB ^1^
Series A	Isoquinoline	Y511 (donor); S512 (donor)
Series B	3-Hydroxy-3,4-dihydroquinolin-2(1*H*)-one	Y511 (donor); S512 (acceptor); R557 (donor).
**C11ak**	1,2,3,4-Tetrahydroquinolin-3-ol	Y511 (donor); S512 (donor); N551 (acceptor). ^2^
**C11al**	2-Oxo-1,2-dihydro-quinoline	Y511 (donor); S512 (donor).
**C11am** and **C36i**	3,4-Dihydroquinolin-2(1*H*)-one	Y511 (donor); S512 (donor).
**C11an**	Indolin-2-one	Y511 (donor); S512 (donor).
**C11ao**	2*H*-benzo[b]-[1,4] oxazin-3(4*H*)-one	Y511 (donor); S512 (donor).
**C36h**	1,2,3,4-Tetrahydroquinolin-3-ol	Y511 (donor); T550 (acceptor). ^3^

^1^ HBs are formed with side chain groups, with the exception of the interaction with T550. ^2^ HB with the CO of N551. ^3^ HB with the backbone CO of T550.

## Data Availability

The data presented in this study are available on request from the corresponding author.

## Supplementary Materials

The following are available online. The zip files SeriesA.zip, SeriesB.zip, and SeriesC.zip contain the docked structures of the compounds from series A, B, and C, respectively, in the mol2 format. Figure S1: Binding modes of 5,5-diarylpentadienamides (DPDAs) and capsazepine as TRPV1 antagonists. Figure S2: Hydrogen bond interactions of the head groups of transient receptor potential vanilloid 1 (TRPV1) modulators with polar residues in the vanilloid pocket. Figure S3: Binding modes of the DPDAs in the TRPV1 structure with the Protein Data Bank (PDB) code 5IRX. Figure S4: Root mean square deviation (RMSD) values that were used to compare the three-dimensional (3D) positions of the neck and head groups of the DPDAs docked inside the structure of the TRPV1 with the PDB code 5IRX. Table S1: Validation statistics for the 2D autocorrelation model with different training–test set splits. Table S2: Random reorganization tests for the 2D autocorrelation model using six replicas.

## Sample Availability

Samples of the compounds are not available from the authors.

## Abbreviations

3D	Three-dimensional
DPDA	5,5-Diarylpentadienamide
GA	Genetic algorithm
HB	Hydrogen bond
IFP	Interaction fingerprint
LOO-CV	Leave-one-out cross-validation
PDB	Protein Data Bank
RMSD	Root mean square deviation
QSAR	Quantitative structure-activity relationship
TRPV1	Transient receptor potential vanilloid 1
