# Supplementary material for: Computational Modeling to Explain Why 5,5-Diarylpentadienamides are TRPV1 Antagonists
Source: Molecules. 2021 Mar 21;26(6):1765. doi: 10.3390/molecules26061765 (PMC8004144; doi:10.3390/molecules26061765)
Supplement: Supplementary file 1 [file molecules-26-01765-s001.zip › molecules-1134342-supplementary-revised/molecules-1134342-supplementary-revised.pdf]

Supplementary material.

Figure S1. Binding modes of DPDAs and capsazepine as TRPV1 antagonists. (a) Binding mode of capsazepine in PDB with code 5IS0. (b) Binding mode of a representative of the compounds from the series A. (c) Binding mode of a representative of the compounds from the series B. (d) Comparison of the binding modes.

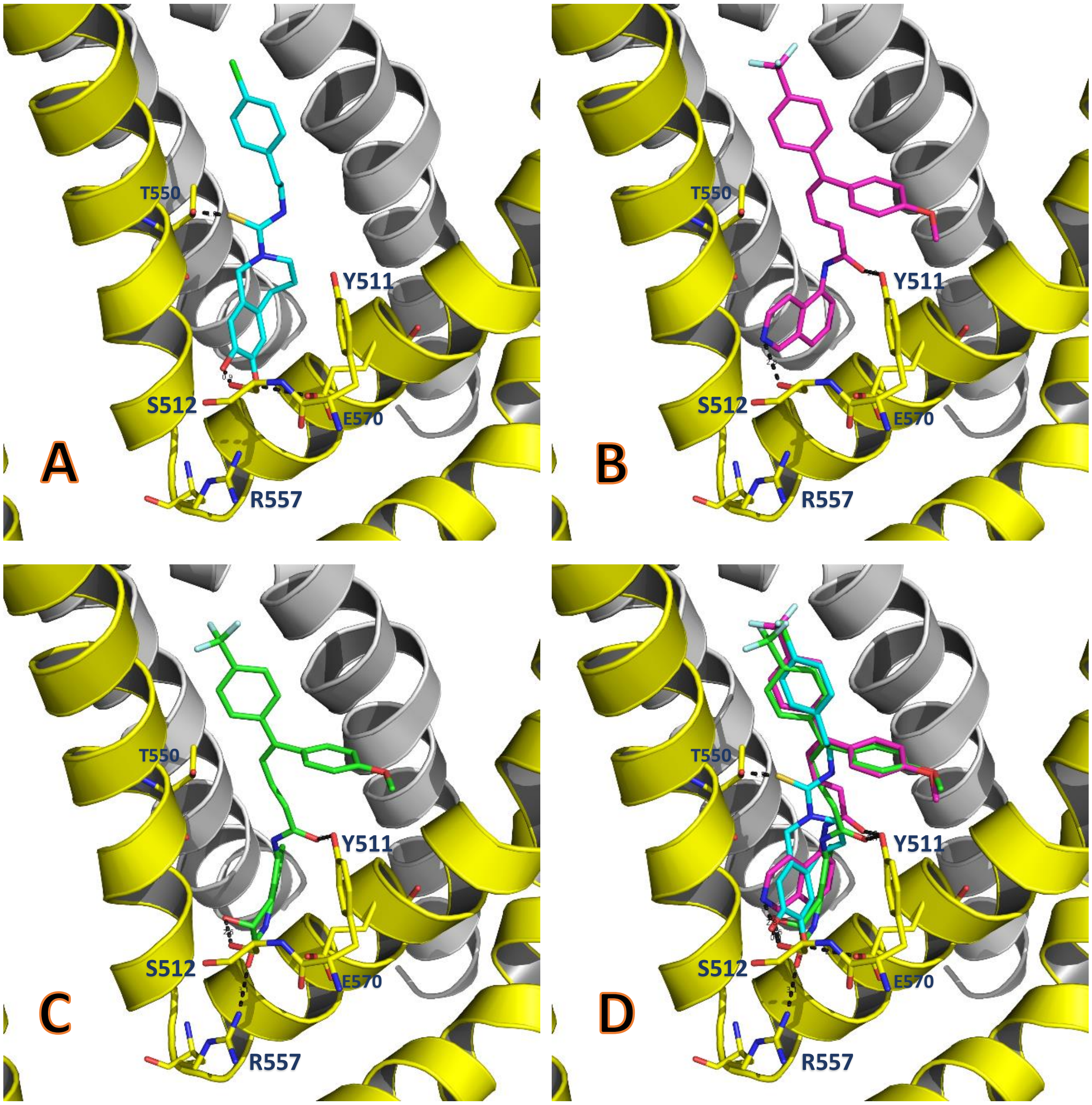

Figure S2. Hydrogen bond interactions of the head groups of TRPV1 modulators with polar residues in the vanilloid pocket. (a) DPDAs from series A, (b) DPDAs from series B, (c) capsazepine in PDB with code 5IS0, (d) resiniferotoxin in PDB with code 5IRX.

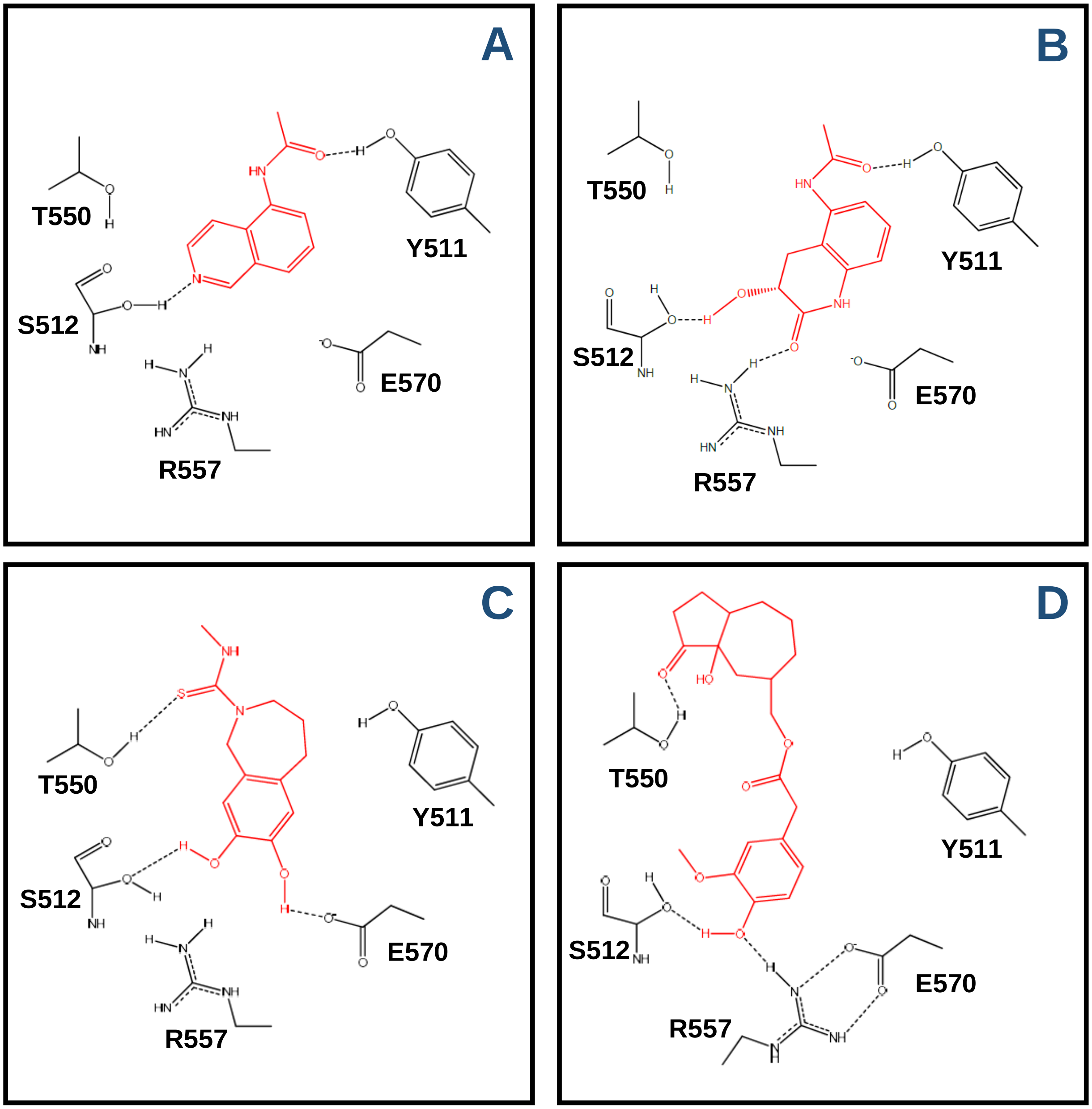

Figure S3. Binding modes of DPDAs in TRPV1 structure with PDB code 5IRX (active conformation). (a) Binding modes of compounds of the series A. (b) Binding modes of compounds of the series B. (c) Binding modes of compounds of the series C. DPDAs are represented in green sticks, chains A and B of TRPV1 are represented in yellow and gray cartoon representations, respectively.

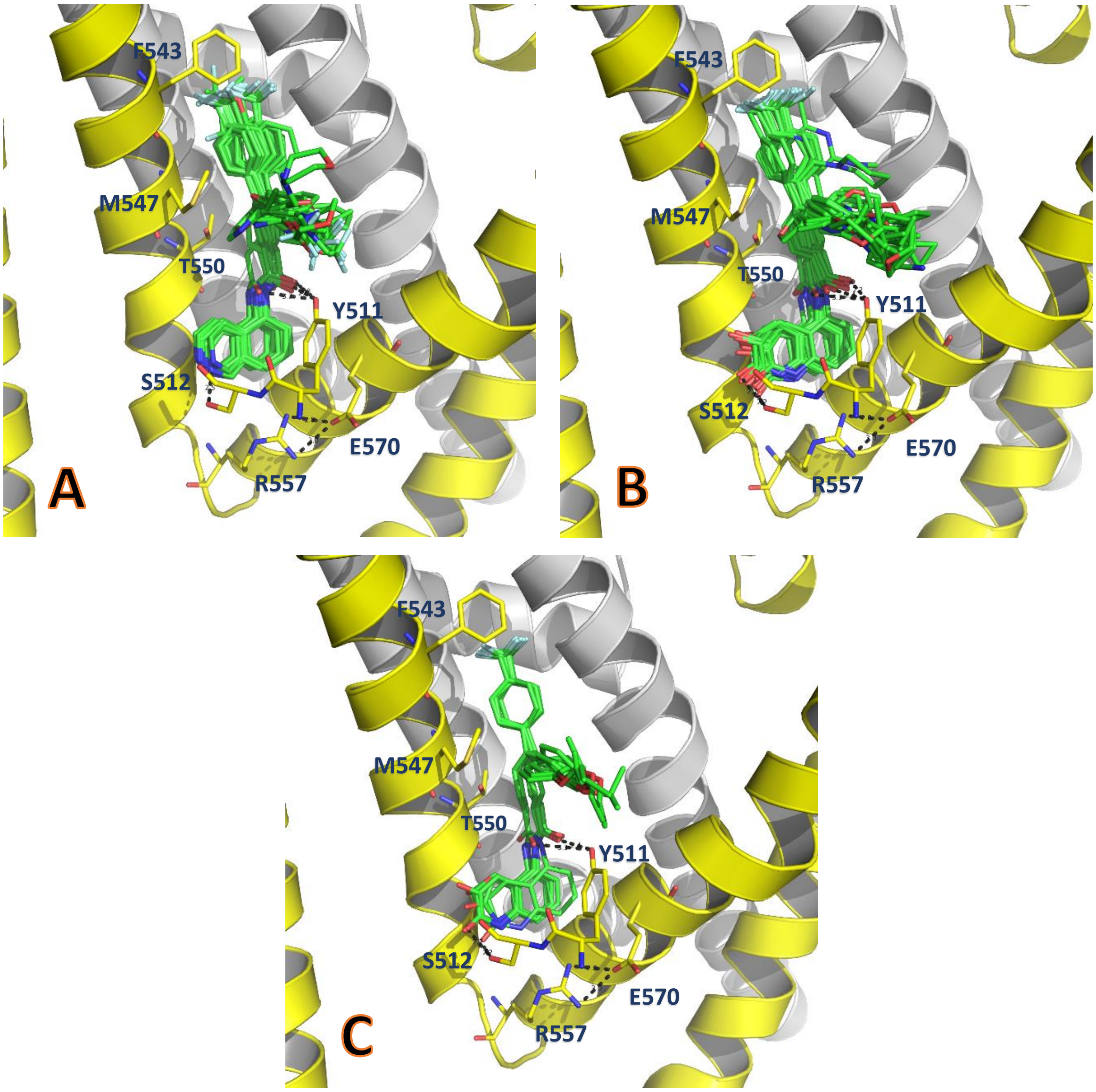

Figure S4. RMSD values to compare the 3D positions of the neck and head groups of the DPDAs docked inside the structure of TRPV1 from PDB with code 5IRX (active conformation). (a) RMSD values between the neck groups of compounds with respect to the neck groups of **A11a** (closed triangle) and **B11u** (open triangle). (b) RMSD values between the head group of compounds from series A with respect to the head group of **A11a**. (c) RMSD values between the head group of compounds from series B with respect to the head groups of **B11u**.

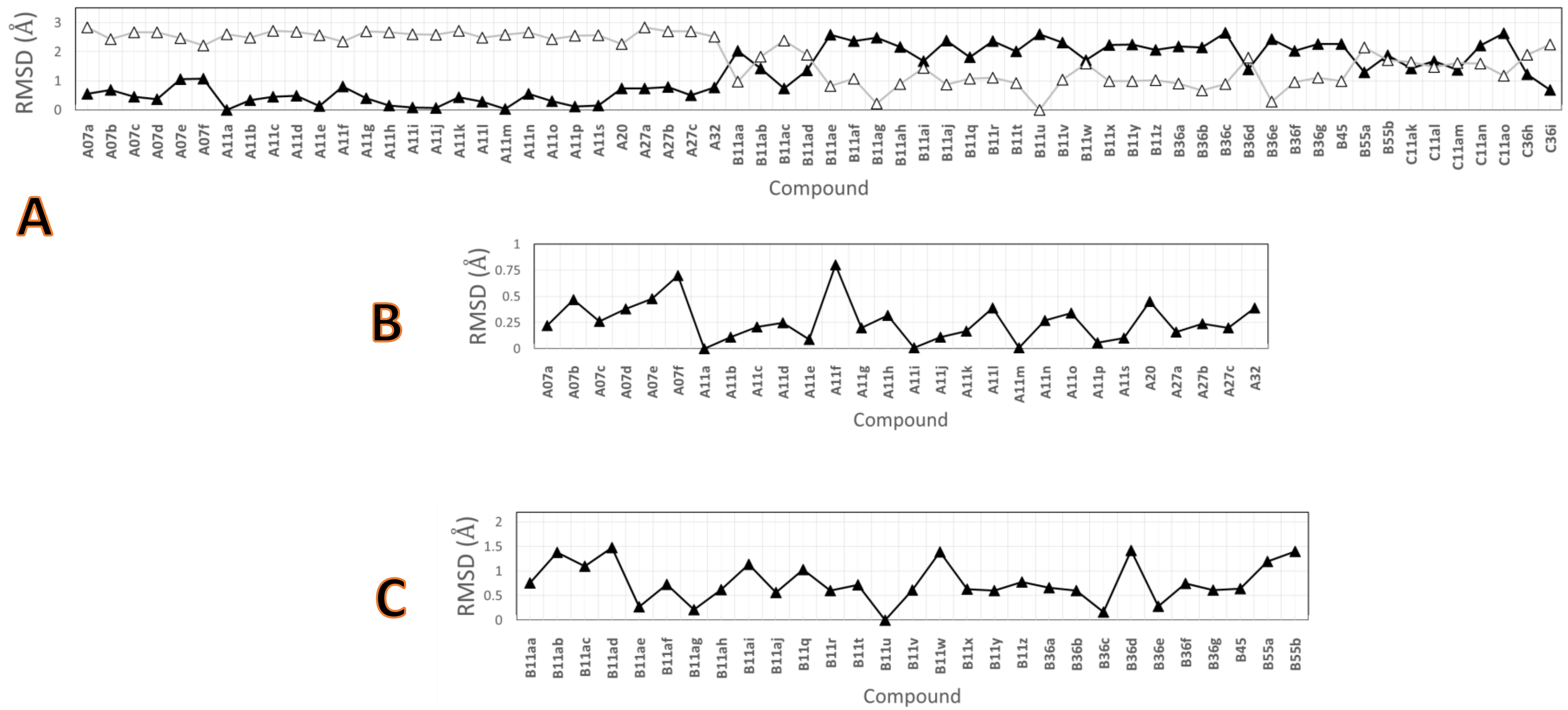

Table S1. Validation statistics for the 2D Autocorrelation model when different training-test set splitting.

Splitting	Test set compounds (training set contains the remaining compounds)	R^2	Q^2	R^2_{test}
1	**A07e**, **A07f**, **A11g**, **A11i**, **A27a**, **B11ab**, **B11ad**, **B11aj**, **B11u**, **B36a**, **C11ao**, **C36i**	0.697	0.592	0.684
2	**A11c**, **A11g**, **A11j**, **A11o**, **A27b**, **B11aa**, **B11ae**, **B11af**, **B11w**, **B11z**, **B36b**, **C11al**	0.695	0.571	0.775
3	**A07d**, **A11g**, **A11i**, **A11n**, **A11p**, **A11s**, **B11ac**, **B11ad**, **B11ae**, **B11t**, **B36d**, **C36i**	0.715	0.606	0.584
4	**A07c**, **A11a**, **A11d**, **A11k**, **A20**, **A27b**, **B11aj**, **B11q**, **B11t**, **B11x**, **B36f**, **C11am**	0.695	0.573	0.662
5	**A07b**, **A11c**, **A11d**, **A11e**, **A11s**, **A27a**, **B11ae**, **B11ai**, **B11r**, **B45**, **B55b**, **C11ak**	0.662	0.536	0.790
6	**A07d**, **A07f**, **A11c**, **A11n**, **A11o**, **A11s**, **B11ad**, **B11ah**, **B11ai**, **B11t**, **B11y**, **B11z**	0.702	0.587	0.743

Table S2. Random reorganization tests for the 2D Autocorrelation model by using six replicas.

Replica	R^2	Q^2	R^2_{test}
1	0.082	-0.366	-0.303
2	0.106	-0.140	-0.020
3	0.025	-0.324	0.030
4	0.185	0.154	0.088
5	0.087	-0.173	-0.010
6	0.046	-0.345	0.003
